# Fifteen per Cent

**Published:** 1890-11-08

**Authors:** 


### Passing Topics.

### FIFTEEN PER CENT.

To those who are interested in charitable work, and a fair proportion of people would consider themselves entitled to be included in this category, there is something startling in the announcement which is appearing in one at least of our leading daily papers:

"The Council of an established charity, under Royal patronage, desires the kind aid of ladies and gentlemen of position to advocate its claims to public support: they would tender to each collector 15 per cent. upon amounts collected towards the unavoidable expenses of collection. All communications will be regarded as strictly confidential, but name and address should be sent to."——

Of course, we do not pretend to such portentous innocence as would enable us to ask seriously—can such things be? On the contrary, we have a clear recollection of rumours of incomes earned sub-rosâ by philanthropic pleaders, just as we are taught to believe that certain impecunious members of the aristocracy are not indisposed to turn their attention to the selling of wines and cigars upon commission or to enter upon any other little commercial venture which promises fair remuneration for their efforts. But what passes the limits of reason, and we may add of decency, is, that the council of any charity, and especially of one boasting the patronage of Royalty, should openly invite so questionable co-operation.

For the honour of our institutions, we prefer to believe that this advertisement must emanate from one of those doubtful organizations which, by an unfortunate oversight, have been accorded a recognition to which their work offers no rightful claim, and on behalf of every *bona-fide* undertaking we must enter a protest against the notion that the ways of this anonymous institution are the ways of legitimate charity. At best the public never distinguishes very readily between begging for self and begging for philanthropic work, and we can imagine few things better calculated to make both collectors and contributors pause than the cynical proposal put forward by the "council of an established charity." We regard it as objectionable when the officials of an institution are remunerated even by moderate per centages upon contributions appealed for, though sometimes they are allowed no choice in the matter, and in opposition to the belief that commissions are necessary inducements to energy, we would offer the undoubted fact that the institutions where commissions rule are by no means always the most prosperous and progressive. But the objection to a deduction for commission grows far more pronounced when the person appealing acts ostensibly, possibly ostentatiously, in a purely honorary capacity, and presses the claims of the institution upon his own personal friends.

No doubt the council have only to advertise freely enough to obtain the assistance they ask for, but we shall be surprised if their unhappy inspiration does not inflict considerable injury upon our struggling institutions. Who, we may ask, with any feelings of self-respect, will be willing to undertake a collection for sweet charity's sake when the world will suspect the price of his sympathy to be fifteen per cent. upon the gross returns? There is an insidious suggestiveness about this advertisement which is calculated to poison the minds of many whose native integrity would have kept them free of suspicion that the kindly energies they have seen displayed by some of their acquaintance in philanthropic work could be induced by a hope of personal gain, and human nature is so constituted that the least-suspecting of the readers of this advertisement will feel its influence most.

One act of satisfaction may be fairly demanded of the advertisers, but, we fear, without avail. It is that they will keep the whole credit of their precious scheme to themselves by publicly announcing the name of their institution; and we would venture to recommend to charities conducted on other and more reputable principles that it might be well to make plain to all who are solicited for help what portion, if any, of the contributions made will be subtracted by way of commission.

Agricola.

